# Applying artificial neural-network model to predict psychiatric symptoms

**DOI:** 10.37796/2211-8039.1149

**Published:** 2022-03-01

**Authors:** Elahe Allahyari, Narges Roustaei

**Affiliations:** a Department of Epidemiology and Biostatistics, School of Health, Social Determinants of Health Research Center, Birjand University of Medical Sciences, Birjand, Iran; b Department of Epidemiology and Biostatistics, School of Health and Nutrition Sciences, Social Determinants of Health Research Center, Yasuj University of Medical Sciences, Yasuj, Iran

**Keywords:** Age, Artificial neural network, Education, Employment status, Gender, Mental disorder, Place of residence

## Abstract

**Introduction:**

Mental disorders result in mental disabilities and discomfort in the affected person as they affect both thinking and behavior. Therefore, more vulnerable people must first be identified to improve the psychological level of society.

**Aim:**

This study aims to determine the importance of gender, employment, education, place of residence, and age in predicting mental disorders using artificial neural network modeling.

**Methods:**

The pattern held between variables in this study will be identified using multilayer feed-forward back-propagation neural networks with five inputs and 10 outputs. To determine the neural network with the least sum of square errors, we evaluated the performance of all neural networks with varying algorithms and different numbers of neurons in the hidden layer. Data were analyzed for 380 people aged 10–82 years using the SPSS software.

**Results:**

The optimal neural network model was effective in predicting mental disorders. In this model, the variables of the place of residence, education, age, gender, and employment were important in fitting the optimal model with 34.08, 20.11, 18.93, 14.55, and 12.33%, respectively. The accuracy rate for the neural network model was 99.2%.

**Conclusion:**

To achieve further results in improving mental health problems, it is better to focus more on employed, rural, and younger people with a non-tertiary education level.

## 1. Introduction

Mental disorders include a wide range of diseases that affect one's thinking and behavior, causing discomfort or disability to the sufferer [1–3]. These include mood disorders, depression, anxiety, obsessive-compulsive disorder, bipolar disorder, schizophrenia, mental retardation, and substance use disorders [1, 4]. According to a World Health Organization (WHO) report, 450 million people worldwide suffered from mental or behavioral disorders in 2001 [4, 5]. In Iran, the prevalence of mental disorders is reported to range between 10.8 and 39.1% [6–8]. Although many factors contribute to mental health disorders [3], they may occur due to genetic, social, and psychological factors and various stressors. One of the causes of mental disorders is industrialization and modern lifestyle, which have harmed people's mental health, so that many people, depending on their social status and living conditions, experience mental disorders regularly [9]. In recent years, various studies have examined the status of mental disorders in terms of age, gender, race, and employment status.

According to the WHO, the overall rate of psychiatric disorders in men and women is almost the same, although there are significant gender differences in patterns of mental illness. Common mental disorders such as depression, anxiety, and physical complaints are more common in women [4, 10]. Approximately 41.9% of neuropsychiatric disorders in women and 29.3% in men could be assigned to depressive disorders [11]. Various studies have examined the age of the onset of mental disorders, showing that mental disorders typically occur in childhood or adulthood. Psychotic disorders rarely occur before the age of 14, but there is a significant increase in prevalence between the ages of 15 and 17. Schizophrenia usually occurs in the age range of 15–35 years [12–14]. Some studies have also shown that the prevalence of mental disorders increases with age. Older people experience cognitive and mood disorders such as dementia and depression [7, 15–17]. Other studies have enquired into the impact of the place of residence and education on mental health [4, 10]. The prevalence of mental disorders is higher in urban than in rural areas. Also, illiterate people or those with low levels of education suffer more from mental disorders than individuals with higher education levels [4, 7]. Employment status is one of the important factors in the incidence of mental disorders so that unemployed people have lower mental health levels than employed people [4, 8]. Among employed people, the prevalence of mental disorders in all groups working in a hospital setting is high due to special working conditions [18–20]. Mental disorders could evaluate using various tools such as the General Health Questionnaire (GHQ), Symptom Checklist-90 (SCL), or diagnostic tools such as the Diagnostic and Statistical Manual of Mental Disorders (DSM) or the International Classification of Diseases (ICD) [6, 21–24].

## 2. Purpose

The increasing prevalence of mental disorders in society due to people's reliance on the modern machinery lifestyle, reduced mobility and increased social pressures can pave the way for the development of practical solutions for controlling and preventing the widespread and negative effects of these diseases on society and empowering communities and individuals to the highest standard of health. Given the importance of this issue, the WHO also supports access to quality and affordable mental health care for more than 100 million people in 12 countries in order to promote mental health by 2023 [25]. However, to plan in this line, it is highly important to identify and predict the factors affecting the development of mental disorders and to determine their priorities. Unfortunately, in most studies in this regard, the concurrent effect of different factors has not been investigated [7, 26, 27]. Due to the interaction between variables, however, psychological studies need to cover the effect of different factors simultaneously. On the other hand, there are several methods of analysis to investigate this issue, among which artificial neural network models have a special position due to the lack of limiting assumptions such as normality and their ability to determine the pattern( s) held between variables [28–30]. Furthermore, the Symptom Checklist-90 Questionnaire (SCL-90) is one of the most widely used screening tools to assess the spot prevalence of possible disorders or suspected mental illnesses between different instruments are established for this purpose [27, 31]. Therefore, the present study built on the artificial neural network as a successful tool in recent decades in estimating and predicting the effect of gender, employment status, education, place of residence, and age in predicting mental disorders by SCL-90.

## 3. Methods

To investigate mental disorders, we first divided each of the cities of Birjand and Mashhad into 4 geographical districts. We then randomly selected 25 households in each district and asked two randomly selected family members to complete the SCL-90 questionnaire and the initial information checklist, which covered age, education, employment status, place of residence, and gender. The individuals were from the general public between 10 and 82 years old and had no psychological tests to diagnose the disease. To perform this cross-sectional study, the code of ethics (Ir.bums.REC.1398.296) was obtained from Birjand University of Medical Sciences Ethics Committee. To collect the questionnaires, we first explained about the research to the subjects, following which informed consent forms were signed by them. If a participant were illiterate, the interviewer completed the questionnaire through interviews with him/her. In the end, 18 incomplete questionnaires were excluded from the study.

The Symptom Checklist-90 Questionnaire (SCL-90) was introduced by Derogatis et al., in 1973, and revised in 1976 based on clinical experience and psychometric analysis [31, 32]. This self-report questionnaire consists of 90 items on a 5-point scale that rates people's distress ranging from 0 (not at all) to 4 (extremely). The inventory items cover nine dimensions: somatization (items 1, 4, 12, 27, 40, 42, 48, 49, 52, 53, 56, and 58), obsession and compulsion (items 3, 9, 10, 28, 38, 45, 46, 51, 55, and 65), interpersonal sensitivity (items 6, 21, 34, 36, 37, 41, 61, 69, and 73), depression (items 5, 14, 15, 20, 22, 26, 29, 30, 31, 32, 54, 71, and 79), anxiety (questions 2, 17, 23, 33, 39, 57, 72, 78, and 80), hostility (questions 11, 24, 63, 67, 74, and 81), phobic anxiety (questions 13, 25, 47, 50, 70, 75, 82 and 86), paranoid ideation (questions 8, 18, 43, 68, 76, and 83), and psychoticism(items 7, 16, 35, 62, 77, 84, 85, 87, 88, and 90). Seven questions, i.e., 19, 44, 59, 60, 64, 66, and 89, are also included in the inventory in addition to the above dimensions, which are clinically important and contribute to the general indicators of the test. These questions are not graded as one of the dimensions of the test but are added to the overall coefficient scores.

### 3.1. Data analysis

The neural network is a parallel processing method that is formed by connecting simple computational units called neurons in the input, middle, and output layers. The input layer neurons are the same as the independent variables, and the output layer neurons are the response variables. Therefore, the number of neurons in the middle layer, the type of connection of neurons in this layer with other layers, and their connection functions are among the parameters that determine the structure of the neural network [33, 34]. In this study, we decided to use multilayer feed-forward back-propagation supervised neural networks, as one of the most widely used and powerful neural networks in predicting and modeling dental anxiety [33, 35]. To this end, we used 90% of the data in the network training process, and the remaining 10%, in model evaluation. To determine the proper connection function, we employed the most widely used functions, i.e., hyperbolic tangent or sigmoid, to connect the input and middle layer neurons as well as linear, hyperbolic tangent or sigmoid functions to connect the middle and output layer neurons [36, 37]. After selecting the appropriate function, we tried to optimize the neural network by increasing the number of neurons in the middle layer. In order to avoid the effect of random assignment of weights and random correlations, we repeated each grid three times and used the average sum of square errors as a suitable indicator in evaluating the model. A total of 346 participants were used in network training, and the remaining 34 participants were employed to test the performance of the model. Information from all records was used in the training dataset with Adam optimizer and Initial Lambda, Initial Sigma, Interval Center, Interval Offset, and Maximum Training Epochs were 5e-7, 5e-5, 0, ±0.5, and automatically, respectively. Lastly, the importance of age, education (tertiary, non-tertiary), employment (unemployed, employed), gender (female, male), and place of residence (urban, rural) in predicting mental disorders were determined. All analyses were performed using the Statistical 100 Package for Social Sciences version 22 (SPSS Inc., Chicago, Illinois, USA).

## 4. Results

The subjects were 380 people (139 males, 241 females) with a mean age of 28.96 ± 11.59 years, most of whom were urban residents (80.5%). Of the subjects, 46.1% were employed, and they were of relatively similar ratios in terms of tertiary/non-tertiary education (64.2% non-tertiary education, 35.8% with tertiary education) (Table 1).

**Fig. 1:**
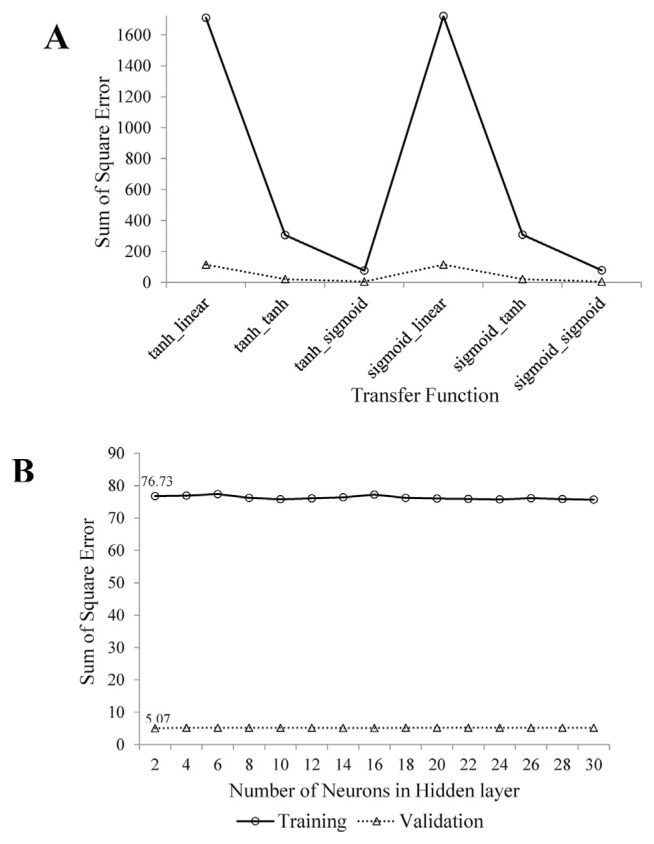
A) The sum of square error of ANN models for different transfer functions; B) The sum of square error of ANN models for different number of hidden neurons.

Figure 1A uses a combination of different functions with 2 neurons in the middle layer to determine the best function for connecting the input, hidden, and output layers in the neural network. As can be seen, the neural network with hyperbolic tangent function to connect the input and middle layers and sigmoid function to connect the middle and output layers had the lowest sum of square errors in both the train and validation sets. The sum of error squares in train and validation sets (76.73 and 5.07, respectively) did not change as the number of neurons in the middle layer increased (Fig. 1. B). Linear correlation between real values and optimal neural network estimates in the dimensions of hostility, anxiety, obsession, interpersonal sensitivity, somatization, psychoticism, paranoid ideation, depression, phobic anxiety, and additional questions were significant (0.13, 0.15, 0.13, 0.15, 0.12, 0.18, 0.20, 0.14, 0.11, and 0.13; p-value <0.05; Table 2).

It should be noted that, there is no complete norm in this test, and a score between 1 and 3 in each dimension usually indicates a morbid state and 3 and above indicates psychosis. However, depression scores between 1 and 2 usually indicate a morbid state and 3 and above indicates psychosis. Anyone shows psychosis stage in any dimension of our study. So, performance of developing ANN model in all 10 subscales of SCL-90 has detection rates higher than 99.2% and false positive and false negative lower than 0.8% (Table 3).

Ultimately, Table 4 shows that from among the studied variables, residence, education, and age with 34.08, 20.11, and 18.93%, respectively, constituted the most important variables, while gender and employment with 14.55 and 12.33%, respectively, were of secondary importance for designing the optimal model. Therefore, the importance of the variables of residence, education, and age in determining the mental disorders of individuals is close to 70%, which indicates the high importance of these variables in overcoming these problems. According to the weights of nodes in the optimal neural network, it is observed that employed, rural, and younger people with a non-tertiary education level experienced more psychological problems in all subscales of the inventory (Table 5). However, in the subscales of psychoticism, phobic anxiety, hostility, and somatization, men suffered from greater disorders than women, whereas in other subscales, women showed more mental disorders.

## 5. Discussion

The aim of this study was to investigate the factors affecting psychiatric disorders simultaneously using an advanced statistical method. Recognizing and prioritizing these factors and developing strategies to improve the mental health of individuals seems essential both at the individual and community levels. According to the literature, psychiatric disorders constitute the fifth cause of disability in people [23, 38].

Based on the results of this study, residence with 34% was the most important factor affecting the modeling of mental disorders in this study. People residing in rural areas experience more mental disorders than urban dwellers. Since agriculture in rural areas is the economic driver of rural development, recent droughts have caused economic stagnation in these areas. Therefore, low economic conditions and unemployment in rural areas can be one of the reasons for the increase in mental disorders in these areas. However, in similar studies, urbanization has been considered as one of the causes of mental disorders [7, 22]. One cause of this discrepancy can be the concurrent consideration of variables and interactions between them.

Education has also been recognized as a primary factor in modeling in this article such that individuals without a tertiary degree had more mental disorders than those who were graduated from a university. Similar studies have reported a higher prevalence of psychological disorders in people with low socioeconomic status, including low income and education [7, 22, 23]. The results of the present study also showed that mental disorders decrease with age and that younger people had more mental disorders. During adolescence, mood swings and confused thoughts, caused possibly by puberty, academic challenges, drug use, etc., have destructive effects and cause people to suffer from a variety of psychological disorders. Alongside this, studies indicate a high prevalence of some mental disorders in adolescence and early adulthood [12, 14]. Therefore, timely interventions may reduce the severity of these disorders in these groups and improve the mental state of society. In this study, as in a study from Brazil, employed people reported more mental disorders than students and housewives [39]. This can indicate the negative effects of workplace stress and job dissatisfaction.

According to the results of this study, women experience different types of mental disorders to a greater extent than men, except for psychoticism, phobic anxiety, hostility, and somatization, which were more common in men than women. In many studies, the prevalence of psychological disorders has been reported more in women than men due to biological causes, environmental stresses, or limited social participation [6, 7, 9, 23, 40, 41]. Moreover, in case of being single, increased age of marriage and increased desire to live as a single person, and in case of being married, increased family quarrels and divorce, a lower spirit of understanding and compromise between couples, and reduced role and influence of family on children, among others, can be the underlying causes of these mental disorders in women.

The present study is important because restricted assumption lack specific assumptions for analysis and its concurrent consideration of factors and their interactions in the model. ANN is an appropriate approach for predicting psychiatric symptoms in general populations. The accuracy rate for ANN model was 99.2% in this study. Similar study showed that accuracy rate was 90.65% and 75.96% for ANN and the logistic regression model, respectively. Also, among demographic characteristics, the level of education was a significant effect on psychiatric symptoms in ANN model. The ANN models versus the univariate models such as the logistic regression models were appeared to be more powerful and flexible in predicting psychiatric symptoms [35].

## 6. Limitation

Our study had some potential limitations. First, few studies have evaluated the psychiatric symptoms of the SCL-90 with ANN, and this made desirable comparisons difficult.

Second, in this study as in most similar studies, due to the difficulty of following people and the sensitivity of the subject, cohort studies and the study of cause and effect relationships were not possible. Third, the self-reporting of information as in all similar survey studies, can affect the accuracy of the results. Therefore, if possible, it is recommended to design prospective studies and investigate the effect of other variables affecting mental disorders in future research.

## 7. Conclusion

According to the findings of this study, in the first level, special attention should be directed to the place of residence, education, and age of individuals. Rural youth who do not have an academic education are among the high-risk groups that are exposed to a variety of mental disorders and should be given more attention. With the increase of urbanization, the tendency of younger people to enter cities has increased and because urbanization also has its own tensions, these disorders get intensified in individuals and will ultimately have bad effects on the community. Creating welfare facilities, employment, and further development of villages can be effective in preventing the increase of urbanization and reducing the mental disorders of people in rural areas.

At the second level, the variables of gender and employment are considered, which should play a major role for women as family educators and the main transmitter of values. The role of mothers in defending national and religious identity and transmitting values is very prominent because all religious, moral, and national values, among others, are formed, institutionalized, and transmitted to society within the family. Special attention should be paid to women in the society. Therefore, having women with mental health in society will lead to the production of a generation with good mental health. Also, necessary measures should be taken to deal with and eliminate stress and job insecurity. Job insecurity causes a person to lose energy and make him/her tired, depressed, and prone to all kinds of mental disorders. Therefore, creating and expanding protective laws concerning different jobs can be helpful and can increase the efficiency and efficacy of employed people.

## Figures and Tables

**Table 1:** Demographic characteristics in the study population.

Variable	N (%)
Gender	
Male	139 (36.6%)
Female	241 (63.4%)
Education	
Non academic	244 (64.2%)
Academic	136 (35.8%)
Employment status	
Unemployed	205 (53.9%)
Employed	175 (46.1%)
Place of residence	
Urban	306 (80.5%)
Rural	74 (19.5%)
Age	28.96 ± 11.59

**Table 2:** Correlation between the predicted values of ANN model and the actual values in 10 subscales of SCL-90.

SCL-90 subscales	Correlation (P-Value)
Psychoticism (P)	0.13 (P = 0.013^a^)
Paranoid Ideation (PI)	0.15 (P = 0.003^a^)
Phobic Anxiety (PA)	0.13 (P < 0.013^a^)
Hostility (H)	0.15 (P = 0.003^a^)
Anxiety (A)	0.12 (P = 0.019^a^)
Depression (D)	0.18 (P < 0.001^a^)
Interpersonal Sensitivity (IS)	0.20 (P < 0.001^a^)
Obsessive Compulsive (OC)	0.14 (P = 0.005^a^)
Somatization (S)	0.11 (P = 0.027^a^)
Additional Scale (AS)	0.13 (P = 0.013^a^)

a Significant correlation at α = 0.05.

**Table 3:** Performance of developing ANN models in 10 subscales of SCL-90.

Subscales	Detection rate	False Positive	False Negative
P	99.48%	0.26%	0.26%
PI	99.2%	0.8%	0%
PA	99.7%	0.3%	0%
H	99.7%	0%	0.3%
A	99.5%	0.5%	0%
D	100%	0%	0%
IS	99.7%	0.3%	0%
OC	99.48%	0.26%	0.26%
S	99.7%	0%	0.3%
AS	99.48%	0.3%	0%

**Table 4:** The variable importance from the selected Artificial Neural Network.

Variables	Importance
Gender	14.55%
Employment status	12.33%
Education	20.11%
Place of residence	34.08%
Age	18.93%

**Table 5:** The weight of trained ANN for 10 subscales of scl-90.

	Hidden layers	

	Node1	Node2
Input layer		
Male	1.099	0.410
Female	−0.114	−0.177
Unemployed	0.344	0.052
Employed	−0.040	0.249
Non academic	−0.208	0.869
Academic	0.535	0.056
Urban	0.640	−0.087
Rural	−0.212	0.700
Age	0.563	−0.050
Output layer		
Psychoticism	−0.169	0.377
Paranoid ideation	−0.256	0.200
Phobic anxiety	−0.099	0.474
Hostility	−0.206	0.401
Anxiety	−0.185	0.303
Depression	−0.362	0.136
Interpersonal sensitivity	−0.539	0.046
Obsessive compulsive	−0.345	0.037
Somatization	−0.089	0.222
Additional scale	−0.305	0.132
